# Frangipani, hibiscus, marigold, and rose leaf imageimagemagei dataset for plant species identification

**DOI:** 10.1016/j.dib.2026.112816

**Published:** 2026-05-06

**Authors:** Md. Mafiul Hasan Matin, Md. Sefatullah

**Affiliations:** a Department of Computer Science and Engineering, Netrokona University, Netrokona 2400, Bangladesh; b Department of Computer Science and Engineering, Daffodil International University, Bangladesh

**Keywords:** Machine learning, Deep learning, Computer vision, Image classification, Convolutional neural networks, Plant species recognition, Agricultural informatics, Botanical image analysis

## Abstract

The Frangipani–Hibiscus–Marigold–Rose (FHMR) Leaf Image Dataset is a curated, field-collected image resource designed to support reproducible research in plant species identification and computer vision–based agricultural applications. The dataset comprises 1080 original leaf images (270 per species) captured from healthy plants in nursery environments, along with 6480 systematically generated augmented images (six per original), resulting in a total of 7560 images. All images are standardized to 512 × 512 pixels in JPEG format. Image acquisition was conducted in Savar, Dhaka, Bangladesh, using an iPhone 14 Pro camera under both neutral and natural background conditions. Augmentation was performed using a deterministic, parameter-bounded pipeline (random seed = 42), incorporating geometric and photometric transformations to enhance variability while maintaining biological plausibility. In addition to the image files, the dataset repository provides a complete metadata manifest, augmentation scripts, and environment specifications to ensure transparency and repeatability. The FHMR dataset is publicly available via Mendeley Data (DOI: 10.17632/tkgbrvb2c2.4) under a CC BY 4.0 license and serves as a benchmark resource for machine learning–based plant species recognition and related computer vision tasks.

Specifications Table

**Table:** 

Subject	Computer Sciences
Specific subject area	Plant Science; Computer Vision; Machine Learning; Agricultural Informatics
Type of data	Image (.jpg)
Data collection	The Frangipani–Hibiscus–Marigold–Rose (FHMR) leaf images were manually collected from healthy ornamental plants in nursery environments under both neutral and natural background conditions. Images were captured using a high-resolution smartphone camera at distances of approximately 20–50 cm and under daylight illumination between 09:00 and 16:00. Each leaf was photographed from multiple viewpoints to capture variability in shape, orientation, and texture. Species identification was performed in-field during acquisition and subsequently cross-validated by a botanically knowledgeable colleague. Ambiguous, occluded, or inconsistent samples were excluded during manual curation.
Data source location	Institution: Diploma Krishibid Nursery and Hossain Nursery Zone: Dattapara, Birulia,Savar Country: Bangladesh Latitude and Longitude: 23° 45′ 17.15″ N, 90° 22′ 34.12″ E
Data accessibility	Repository name: Mendeley Data Data identification number: 10.17632/tkgbrvb2c2.4 Direct URL to data: https://data.mendeley.com/datasets/tkgbrvb2c2/4
Related research article	None

## 1 Value of the Data

• The FHMR Leaf Image Dataset provides naturally-acquired leaf images of four widely cultivated ornamental species—Frangipani, Hibiscus, Marigold, and Rose—supporting research in plant identification and botanical image analysis [[1], [2], [3]].
• Typical use cases include species classification benchmarking, evaluation of augmentation strategies, and transfer learning experiments [4]. In addition to plant species classification, the FHMR Leaf Image Dataset can support downstream computer vision applications such as plant disease detection, mobile-based plant recognition systems, smart agriculture tools, biodiversity monitoring, and transfer learning research. The dataset may also be useful for developing edge-AI applications for smartphone-based plant identification in nursery and agricultural environments.
• Images were captured under diverse lighting conditions and backgrounds, allowing development of models robust to real-world variability.
• The dataset can be used to train, validate, and benchmark machine learning and deep learning models (e.g., ResNet, VGG, EfficientNet), enabling reproducible comparative studies. For reproducible benchmarking, a standard dataset split of 70% training, 15% validation, and 15% testing is recommended. It is advised to use only original images for validation and testing to avoid data leakage.
• The dataset is released under a CC BY 4.0 license, promoting open and reproducible research. For unbiased evaluation, users are encouraged to test models using original (non-augmented) images [[5], [6], [7]].

## 2 Background

The Frangipani–Hibiscus–Marigold–Rose (FHMR) Leaf Image Dataset was developed to support research in automated plant identification and classification using computer vision and machine learning methodologies. As AI-based approaches become increasingly integrated into agriculture, ecology, and botanical research, there remains a strong need for high-quality, labeled datasets that reflect real-world leaf morphology variations across species. Many existing public datasets either focus on a narrow selection of plants or lack sufficient diversity in image capture conditions [8]. The FHMR dataset helps address these limitations by providing clearly labeled leaf images from four widely grown ornamental plant species, photographed under varying lighting, viewpoints, and environmental backgrounds. Images were captured in nursery settings to ensure accurate representation of natural leaf structures, aiding their use in training and evaluating plant identification models. To contextualize this contribution, we include a brief comparison with established datasets: the Oxford 102 Flowers dataset [9] emphasizes images of flowers sourced from the web rather than field photography of leaves, while the PlantVillage dataset [10] features many agriculturally important species but focuses primarily on leaf disease classification rather than ornamental plant differentiation. The FHMR dataset thus provides complementary value by offering leaf-based, captured in the field imagery from commonly cultivated tropical ornamentals frequently used in landscaping and botanical studies in Bangladesh. Although narrower in taxonomic breadth, the dataset delivers high-quality, real-world leaf imagery specifically suited for species-level identification tasks based on morphological characteristics. Compared to large-scale datasets, FHMR provides controlled, field-acquired leaf images with consistent labeling and real-world variability, making it suitable for focused benchmarking and transfer learning tasks.

## 3 Data Description

The FHMR Leaf Image Dataset consists of leaf images from four flower plant species: Frangipani, Hibiscus, Marigold, and Rose. The dataset includes 1080 original images and 6480 augmented images, resulting in a total of 7560 images. All images are provided in JPEG format and resized to a uniform resolution of 512 × 512 pixels. Original images were captured at native resolutions ranging from 800 × 600 to 1920 × 1080 pixels and subsequently resized for consistency.

Among the original images, 640 were captured against neutral backgrounds and 440 against natural backgrounds. This background distribution, along with other acquisition details, is explicitly documented in the accompanying metadata manifest. Augmented images were generated from the original set using controlled geometric and photometric transformations to increase intra-class variability while preserving species identity.

The dataset is organized into a hierarchical folder structure for easy access and reproducible experimentation. At the top level, the dataset contains separate folders for Original_Images, Augmented_Images, Metadata, and Scripts. Within the Original_Images and Augmented_Images folders, subfolders are provided for each species: Frangipani, Hibiscus, Marigold, and Rose. Additional train, validation, and test split folders can be generated using the metadata manifest file, which specifies the assigned split for every image.

**Fig. 1:**
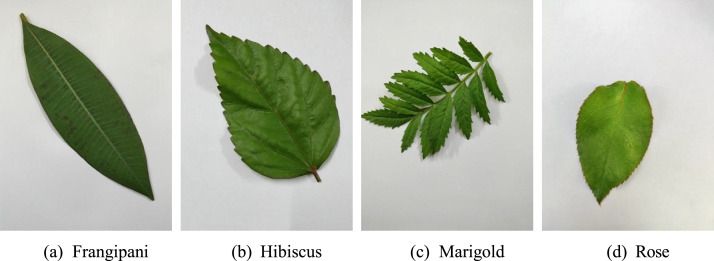
Representative leaf images from the four plant species included in the dataset: (a) Frangipani leaf – elongated, glossy, dark green with smooth edges; (b) Hibiscus leaf – broad, ovate, medium green with serrated margins; (c) Marigold leaf – pinnate, light green with narrow leaflets and jagged edges; and (d) Rose leaf – compound, dark green with pointed leaflets and serrated margins. The selected examples also demonstrate variation in leaf orientation, lighting condition, leaf maturity, and background complexity.

**Fig. 2:**
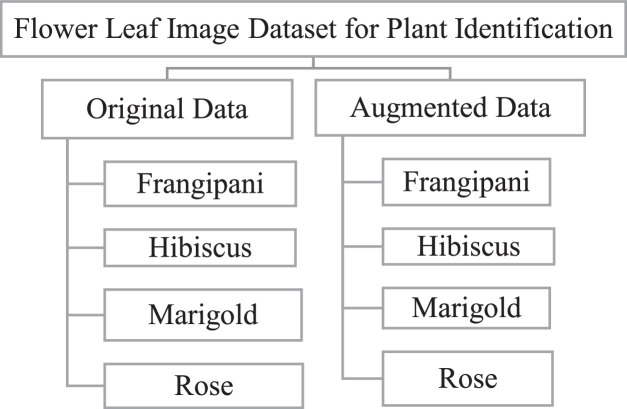
Hierarchical folder organization of the FHMR Leaf Image Dataset showing separate directories for original images, augmented images, metadata files, and preprocessing scripts.

Filenames follow a standardized naming convention that includes the species name and a unique identifier, ensuring traceability between original and augmented samples. For example, original images use names such as Frangipani(1).jpg, Hibiscus(25).jpg, and Rose(143).jpg, while augmented images use names such as Aug_Frangipani(1).jpg and Aug_Rose(143).jpg. This naming structure allows users to easily identify the source species and distinguish between original and augmented images. Table 1 summarizes the dataset statistics by species, while Fig. 1 presents representative sample images. The overall folder hierarchy of the dataset is illustrated in Fig. 2.

**Table 1:** Statistics of the flower leaf image dataset for plant identification.

Folder Name	Original Images	Augmented Images	Total Images
Frangipani	270	1620	1890
Hibiscus	270	1620	1890
Marigold	270	1620	1890
Rose	270	1620	1890
**Total**	**1080**	**6480**	**7560**

The dataset includes a manifest CSV file that provides structured metadata for each image. The metadata fields are summarized in Table 2. The manifest file enables reproducible dataset loading, filtering, and controlled experimental benchmarking.

**Table 2:** Metadata fields included in the FHMR manifest file.

Field Name	Description	Data Type
image_id	Unique identifier for each image	String
label	Plant species (Frangipani / Hibiscus / Marigold / Rose)	String
split	Dataset split (Train / Validation / Test)	String
augmented	Indicates whether the image is original or augmented	String
file_path	Relative file path of the image	String
width	Image width in pixels	Integer
height	Image height in pixels	Integer
channels	Number of color channels (3 for RGB)	Integer
format	Image file format (JPG)	String
color_profile	Color profile information (sRGB)	String
image_type	Indicates whether the image is original or augmented	String
augmentation_method	Type of augmentation applied to the image	String
background_type	Neutral or natural background	String
capture_time	Approximate capture period (morning, noon, afternoon)	String
capture_condition	Lighting condition during image acquisition	String

## 4 Experimental Design, Materials and Methods

### 4.1 Image acquisition

**Fig. 3:**
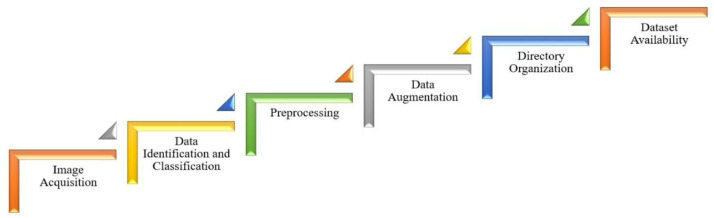
Workflow illustrating the step-by-step process for generating the Flower Leaf Image Dataset for Plant Identification.

Leaf images for the FHMR dataset were acquired in nursery environments and processed to produce a uniform, well-labeled dataset suitable for machine learning applications. Fig. 3 follows a fixed sequence: (i) quality inspection and removal of blurred or duplicate images; (ii) optional center cropping to a square format; (iii) resizing to 512 × 512 pixels using bicubic interpolation; and (iv) data augmentation applied only after preprocessing. All numerical parameters, software tools, and random seeds have been reported to ensure full replicability. The dataset was collected at Diploma Krishibid Nursery and Hossain Nursery in Dattapara, Birulia, Savar, Dhaka, Bangladesh (23°45′17.15″N, 90°22′34.12″E). Images were captured exclusively using the iPhone 14 Pro, which features a 48-MP quad-pixel wide camera with Phase Detection Autofocus (PDAF), Focus Pixels, and second-generation sensor-shift optical image stabilization (OIS). Each leaf was captured from multiple angles at an approximate distance of 20–50 cm using handheld shooting. Image acquisition was performed under varying daylight conditions between 09:00 and 16:00, including bright sunlight, mild shadow, cloudy illumination, and diffused natural light. Leaves were photographed at different maturity stages, sizes, orientations, and viewing angles to capture realistic intra-species variation. Multiple samples of each species were collected from different plants to improve dataset diversity and reduce sampling bias. Some images also include natural nursery backgrounds such as soil, grass, pots, and surrounding vegetation. Neutral backgrounds were used where feasible, while a subset of samples includes natural backgrounds to enhance robustness in downstream machine learning tasks. To ensure privacy and prevent location disclosure, all EXIF metadata, including GPS coordinates, were removed from the images prior to dataset publication. The accompanying metadata manifest CSV retains relevant information necessary for research purposes, such as image_id, label, dataset split (Train/Validation/Test), image dimensions (width, height), number of channels, format, color_profile, and whether the image is augmented. This approach ensures that the dataset remains fully usable for machine learning and computer vision tasks while protecting sensitive acquisition information. Species labeling was initially performed by the authors during image acquisition. To ensure label quality, a botanically knowledgeable colleague independently verified a random subset of the collected images. Disagreements or ambiguous cases were discussed and resolved by consensus, and images deemed inconsistent or unclear were removed during manual curation.

### 4.2 Data augmentation

**Fig. 4:**
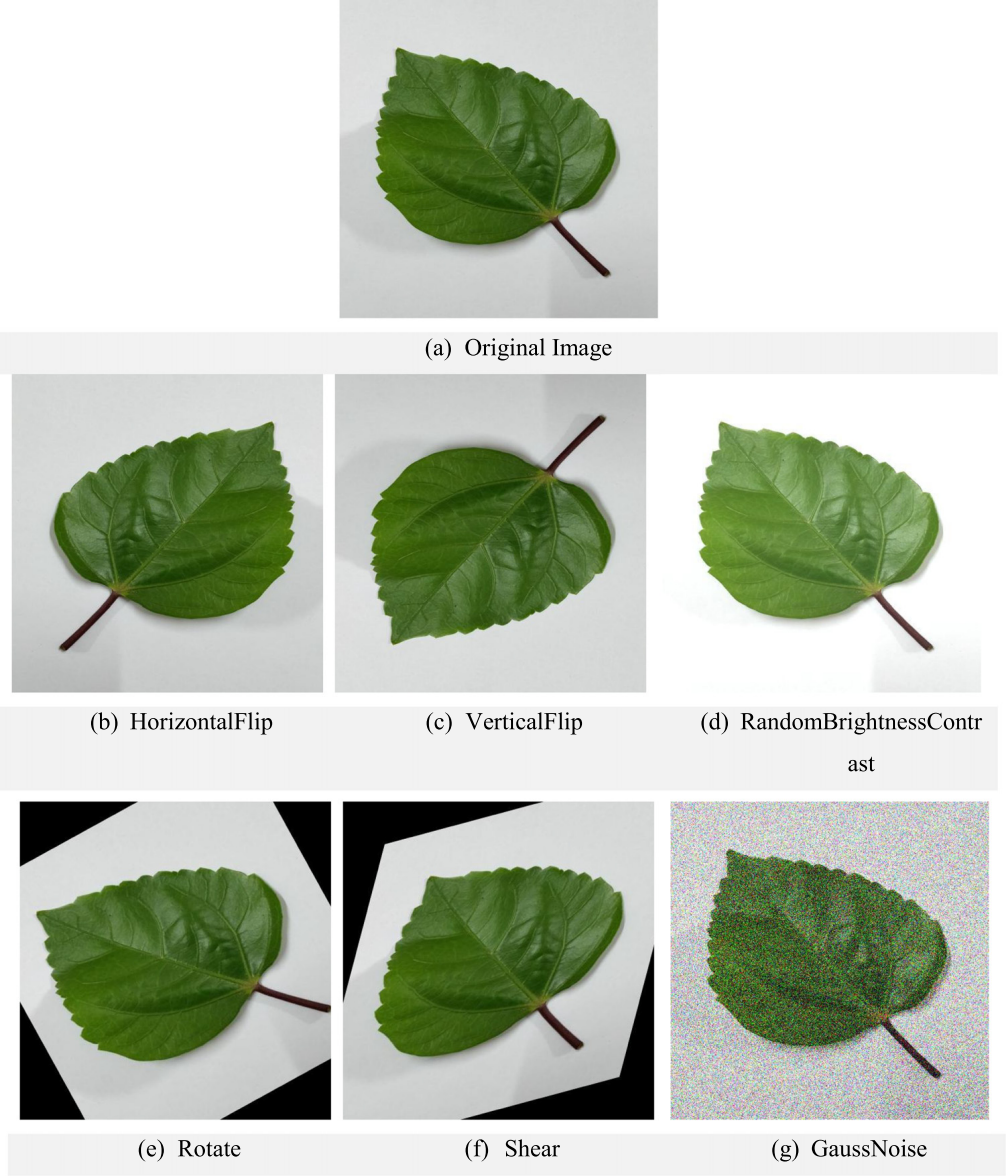
Examples of augmented images illustrating the different transformations applied to the original dataset, including horizontal flip, vertical flip, brightness adjustment, rotation, shear, and Gaussian noise.

Preprocessing was performed before augmentation, including resizing and optional cropping. The augmentation step was designed to generate six augmented images per original to enhance dataset diversity, resulting in 1620 augmented images per class from 270 originals. The augmentation pipeline, applied in random order for each sample, included: random rotation (−30° to +30°), horizontal flip (p = 0.5), vertical flip (p = 0.2), random zoom/scale (0.9–1.1), random translation (±10% of image size), shear (±10°), brightness and contrast adjustments (0.8–1.2), color jitter (saturation factor 0.9–1.1), Gaussian noise (p = 0.2, σ ≤ 0.01), and random cropping + resize (85–100% crop scaled back to 512 × 512). Transformations were combined stochastically to maximize variability, producing six distinct augmented images per original. All augmented images were saved in JPEG format, and Fig. 4 illustrates the six augmentation operations applied to the original images and Table 3 shows the detailed augmentation with parameters and probabilities [11]. Data augmentation was implemented using the Albumentations library (1.4.6), ensuring reproducible, parameter-bounded transformations. The dataset is now available in the Mendeley Data repository [12].

**Table 3:** Summary of augmentation operations applied to the FHMR Leaf Image Dataset.

Operation	Parameter / Range	Probability
Rotation	−30° to +30°	1.0
Horizontal flip	—	0.5
Vertical flip	—	0.2
Scale / Zoom	0.9 — 1.1	0.5
Translation	±10%	0.5
Shear	±10°	0.3
Brightness adjustment	factor 0.8 — 1.2	0.7
Contrast adjustment	factor 0.8 — 1.2	0.7
Hue/Saturation jitter	small	0.5
Gaussian noise	σ ≤ 0.01	0.2
Random crop	area 85–100%	0.5

To ensure full reproducibility, the exact augmentation scripts, and software environment specifications are provided as supplementary materials in the Mendeley Data repository. The code includes the complete augmentation pipeline with fixed random seed (42), as well as a requirements.txt file documenting all Python packages and versions used. These resources allow independent researchers to reproduce the augmented dataset or adapt the pipeline for related plant image datasets.

### 4.3 Camera specification

Images were captured using the iPhone 14 Pro, which features Apple’s advanced computational photography system integrating Phase Detection Autofocus (PDAF), Focus Pixels, and machine-learning–enhanced image processing for high-precision and rapid focusing performance. The device includes a triple-camera system consisting of a 48-megapixel main sensor (wide), an ultra-wide lens, and a telephoto lens, enabling flexible image capture in diverse environmental settings commonly found in nursery environments.

The main (wide) camera uses a 24 mm equivalent lens with an f/1.78 aperture, second-generation sensor-shift optical image stabilization (OIS), and a quad-pixel sensor that supports high-detail imaging. The ultra-wide lens (13 mm, f/2.2, 120° field of view) provides a wider scene coverage that was useful when capturing leaves with natural backgrounds. The telephoto lens (77 mm, f/2.8) offers 3× optical zoom and up to 15× digital zoom, allowing images to be taken from varying distances while preserving clarity, though digital zoom may introduce minor detail loss. All images were captured in a 4:3 aspect ratio and later resized to 512 × 512 pixels during preprocessing.

The wide and telephoto lenses feature Optical Image Stabilization, minimizing blur from hand movement, especially in shaded nursery areas. The iPhone 14 Pro’s computational features such as Smart HDR 4, Deep Fusion, and Photonic Engine further enhance texture detail, color accuracy, and low-light performance. Table 4 summarizes the camera specifications used for dataset image acquisition.

**Table 4:** Specifications of the iPhone 14 Pro camera system used for dataset image acquisition.

Specification	Details
Device Model	iPhone 14 Pro
Camera Setup	Triple-lens system (Main/Wide, Ultra-Wide, Telephoto)
Main (Wide) Lens	24 mm, 48 MP, f/1.78 aperture, 2nd-gen Sensor-Shift OIS, Quad-Pixel Sensor
Ultra-Wide Lens	13 mm, f/2.2 aperture, 120° field of view
Telephoto Lens	77 mm, f/2.8 aperture, 3× optical zoom, up to 15× digital zoom
Focus Technology	Phase Detection Autofocus (PDAF), Focus Pixels
Image Stabilization	Sensor-Shift OIS (wide), Optical OIS (telephoto)
Image Format	JPEG (.jpg)
Aspect Ratio	4:3
Captured Resolution	Native resolution (48 MP) downsampled (resized to 512 × 512 px)
Lighting Conditions	Natural daylight under controlled nursery conditions
Computational Features	Smart HDR 4, Deep Fusion, Photonic Engine, Night Mode

### 4.4 Deep learning model validation

To demonstrate the usability of the dataset, a simple baseline experiment was conducted using a pre-trained CNN model (e.g., ResNet18). The model was trained on 70% of the original dataset and evaluated on 15% validation and 15% test sets. The model achieved an accuracy of approximately 92%, indicating that the dataset is suitable for plant species classification tasks. This result further demonstrates that the dataset can support downstream transfer learning and mobile plant recognition applications.

## Limitations

Despite its usefulness, the dataset has several limitations. First, it includes only four ornamental plant species, which may limit generalization to broader plant classification tasks. Second, all images were collected from a specific geographic region (Savar, Dhaka, Bangladesh), introducing potential geographic bias. Third, image acquisition was primarily performed using a single device (iPhone 14 Pro), which may introduce device-specific characteristics. Future work may address these limitations by including more species, diverse locations, and multiple imaging devices.

## Ethics Statement

The authors confirm that they have read and comply with the ethical requirements for publication in *Data in Brief*. This work does not involve human subjects, animal experiments, or data collected from social media platforms. All plant images were collected from nurseries with permission, and no ethical approval was required for this type of observational data collection.

## Credit Author Statement

**Md. Mafiul Hasan Matin:** Conceptualization, Methodology, Software, Writing – review & editing, Supervision; **Md Sefatullah:** Data curation, Validation, Formal analysis, Visualization;

## Funding

This research did not receive any specific grant from funding agencies in the public, commercial, or not-for-profit sectors.

## Data Availability

The FHMR Leaf Image Dataset is openly available on Mendeley Data at DOI: 10.17632/tkgbrvb2c2.4. The repository includes the complete image dataset (original and augmented), a full metadata/manifest CSV, augmentation and preprocessing scripts, and environment specification files. The dataset is distributed under the Creative Commons Attribution 4.0 International (CC BY 4.0) license.
